# Knowledge, attitude, and perception regarding COVID-19-related prevention practice among residents in Vietnam: a cross-sectional study

**DOI:** 10.3389/fpubh.2023.1100335

**Published:** 2023-06-15

**Authors:** Thoa Le, Trang T. B. Le, Le Van Truong, Mai Ngoc Luu, Nguyen Tran Minh Duc, Abdelrahman M. Makram, Truong Van Dat, Nguyen Tien Huy

**Affiliations:** ^1^University of Medicine and Pharmacy at Ho Chi Minh City, Ho Chi Minh City, Vietnam; ^2^Traditional Medicine Hospital of Ministry of Public Security, Hanoi, Vietnam; ^3^Department of Internal Medicine, University of Medicine and Pharmacy at Ho Chi Minh City, Ho Chi Minh City, Vietnam; ^4^Faculty of Medicine, University of Medicine and Pharmacy at Ho Chi Minh City, Ho Chi Minh City, Vietnam; ^5^School of Public Health, Imperial College London, London, United Kingdom; ^6^School of Tropical Medicine and Global Health, Nagasaki University, Nagasaki, Japan

**Keywords:** knowledge, attitude, perception, practice, COVID-19, Vietnam

## Abstract

**Background:**

Vietnam was one of the countries pursuing the goal of “Zero-COVID” and had effectively achieved it in the first three waves of the pandemic. However, the spread of the Delta variant was outbreak first in Vietnam in late April 2021, in which Ho Chi Minh City was the worst affected. This study surveyed the public's knowledge, attitude, perception, and practice (KAPP) toward COVID-19 during the rapid rise course of the outbreak in Ho Chi Minh City.

**Methods:**

This cross-sectional survey was conducted from 30th September to 16th November 2021, involving 963 residents across the city. We asked residents a series of 21 questions. The response rate was 76.6%. We set *a priori* level of significance at α = 0.05 for all statistical tests.

**Results:**

The residents' KAPP scores were 68.67% ± 17.16, 77.33% ± 18.71, 74.7% ± 26.25, and 72.31% ± 31, respectively. KAPP scores of the medical staff were higher than the non-medical group. Our study showed positive, medium–strong Pearson correlations between knowledge and practice (*r* = 0.337), attitude and practice (*r* = 0.405), and perception and practice (*r* = 0.671; *p* < 0.05). We found 16 rules to estimate the conditional probabilities among KAPP scores via the association rule mining method. Mainly, 94% confident probability of participants had {Knowledge=Good, Attitude=Good, Perception=Good}, as well as {Practice=Good} (in rule 9 with support of 17.6%). In opposition to around 86% to 90% of the times, participants had levels of {Perception=Fair, Practice=Poor} given with either {Attitude=Fair} or {Knowledge=Fair} (according to rules 1, 2, and rules 15, 16 with a support of 7–8%).

**Conclusion:**

In addition to the government's directives and policies, citizens' knowledge, attitude, perception, and practice are considered one of the critical preventive measures during the COVID-19 pandemic. The results affirmed the good internal relationship among K, A, P, and P scores creating a hierarchy of healthcare educational goals and health behavior among residents.

## Introduction

The COVID-19 pandemic has gone through four waves in Vietnam from 23rd January 2020 till now. Vietnam was one of the countries pursuing the goal of “Zero-COVID” and had effectively achieved it in the first three waves of the pandemic. However, like numerous countries in the world, Vietnam labored to prevent the spread of the Delta variant, which broke out first in Vietnam in late April 2021 (1–3). The fourth wave caused many deaths and infected cases in large-scale nationwide areas, especially in the northern areas, in which Ho Chi Minh City was the worst-hit area. There were 328,659 new confirmed cases and 8,237 new deaths reported in only September 2021 (4). Consequently, the case-fatality ratio of Vietnam reached 2.50%, higher than the average global number of 2.06% (5).

Government policies related to COVID-19 eradication, preparedness of healthcare workers, and community awareness of pandemic fighting were notable concerns. One cross-sectional survey conducted across 57 countries, including Vietnam, showed that nurses and doctors who participated in COVID-19 training courses had a “great extent of confidence” in handling suspected COVID-19 patients (6). In addition, an analysis across fourteen countries revealed that early centralized isolation of all confirmed cases without the requirement of the lockdown of an entire city or public areas was a core intervention in the successful control of an outbreak in the early stage, that is when the total number of cases was fewer than 100 (7).

Other than the preparedness of healthcare workers during the COVID-19 pandemic, Yang et al. revealed that gender and occupation were associated with knowledge, while occupation and family economic levels mainly influenced attitude (8). Finally, place of residence, occupation, general knowledge, and attitude determined practice (8). According to a survey of Saudi Arabia's population, older adults were more likely to have a more favorable knowledge, attitude, and practice (KAP) score than younger people (9). Moreover, the KAP scores for women were higher compared to those for men (9). There was also a survey from US households that reported that a low level of knowledge was associated with low education, low income, and age of 35–49 years (10).

The rationale for the application of the KAP study is derived from the principles of health behavior and its typologies (11), which is conceptually operationalized by the health belief model (HBM) of Rosenstock (12) and Janz and Becker (13), a behavior diagnostic framework in health promotion research that is premised on the theoretical links in the constructs of knowledge, attitudes, and perceptions in regard to a risk behavior/practice rather than just knowledge, attitudes, and practice (11–13). In addition, in 1956, Bloom and other psychologists developed a hierarchy of educational goals, which was originally known as Bloom's Taxonomy (14). It included three domains, namely, cognitive (i.e., knowledge), affective (i.e., attitude), and psychomotor (i.e., skills); in particular, there were six major categories of the cognitive domain (i.e., knowledge, comprehension, application, analysis, synthesis, and evaluation) that were reformed over time by many authors (15, 16).

Governments have put in place various measures to address the spread of COVID-19. Although these actions can effectively limit the transmission of the virus, citizens must voluntarily cooperate with these measures for them to be effective. However, compliance with these preventive measures is not universal. Some studies have identified factors that influence compliance with preventive behaviors (17, 18). It has been observed in existing studies that mental health problems and anxiety are frequently experienced during epidemics and that these psychological conditions can impact compliance with preventive behaviors (17, 19, 20). Individuals with higher levels of anxiety tend to exhibit lower levels of compliance with these behaviors (17, 21).

Even though the Vietnamese government had successfully increased the awareness of the population toward COVID-19 by various means, the massive surge of cases in the fourth wave still occurred (1, 22, 23). The cause might have been either the non-compliance in the population or inappropriate restriction policies by the government that became a controversial topic during the COVID-19 pandemic. We hypothesize that the knowledge, attitude, perception, and practice (KAPP) of the population can highly affect the efforts of controlling the spread of the disease. Therefore, this cross-sectional study aimed to contribute to a comprehensive understanding of why Vietnam failed to control by taking into account aspects of knowledge, attitude, perception, and practice (KAPP) toward COVID-19 of Vietnamese residents in Ho Chi Minh City during the rapid rise period of the outbreak.

## Materials and methods

### Study design and participants

The reporting of this study followed the Consensus-Based Checklist for Reporting of Survey Studies (CROSS) guidelines (24). The CROSS checklist was specifically developed to address the unique reporting challenges of survey studies. The checklist is available in Supplementary Table 1. This cross-sectional survey-based study was conducted to assess the awareness of residents toward COVID-19 via KAPP score evaluation in Ho Chi Minh City, Vietnam, from 30th September to 16th November 2021, during the period of the most prolonged city-wide lockdown due to the fourth wave of the COVID-19 pandemic. The online survey was snow-balled to participants through the Zalo app with identified Groups Messenger that we most widely used to manage residents indirectly during the COVID-19 pandemic through every ward level and district level. The inclusion criteria of the participants were residents aged 18 years and older living in Ho Chi Minh City, regardless of their infectious state to COVID-19. Participants who missed more than 50% of the questions were excluded from the study.

All participants had to sign the informed consent embedded in the questionnaire, in which they agreed to participate voluntarily with the right to withdraw at any time and were guaranteed of being anonymous and kept confidential (Appendix 1). The data were extracted and encrypted for analysis.

### Study questionnaire

The study questionnaire contained 21 survey questions divided into five parts (Appendix 1), which were developed on the survey monkey interface (www.surveymonkey.com). The first part assessed the participants' sociodemographic characteristics, including age, gender, occupation, living location, religion, ethnicity, marital status, level of education, and source of information. The second part obtained their knowledge, through four questions, particularly in transmission routes, COVID-19 clinical symptoms, high-risk group evaluation, and spread of virus infection. The third part was about their attitudes toward the “9K message,” which was advanced from the “5K message,” issued by the Vietnamese Ministry of Health regarding measures to prevent COVID-19 infection in the community, while the fourth part determined their perceptions about measures that help prevent the spread of COVID-19 or help isolation at home (25, 26). The final part assessed the practice among residents during the COVID-19 pandemic over the past few days and also their recent difficulties to apply measures. The questionnaire was primarily pretested on 10 individuals to check the clarity and comprehension of all survey questions. The questionnaire was then piloted on 30 respondents to evaluate its reliability. We evaluated the reliability of the questionnaire using Cronbach's alpha coefficient. The Cronbach's alpha coefficient for the overall questionnaire was 0.911, which indicates excellent internal consistency. Modifications were subsequently made if needed. The data collected from the pilot survey were omitted from the final analysis.

The knowledge scale was surveyed with four questions in the form of multiple-choice questions. Each correct choice gained 1 point, while each inaccurate response was deducted 1 point. The maximum total score on the knowledge scale was 19 points. One question contained 10 sub-questions related to attitude, which used the 5-item-Likert scale counting from 1 to 5 points corresponding from strongly disagree to strongly agree. There was a maximum of 50 points on the attitude scale. In terms of perception, the question with a correct response will be added one mark, whereas one with a wrong answer will be subtracted one mark or with no change for “no” or “I don't know” in the given answer. The maximum total score for the perception scale was 14 points. There were 11 sub-questions in the aspect of practice, equivalent to a maximum of 11 scores, and also contained two more questions about some difficulties in applying prevention measures in recent days. After scoring, we converted all 4 scales to a 100-point scale.

### Statistical analysis

All analyzes were performed using R version 4.1.0 software on the Windows 11 platform using the compareGroups, cowplot, and ggplot2 packages.

Sample size calculation: An estimated sample size was based on a single population proportion formula *N* = (Z_α_/2)^2^^*^p^*^(1-p)/MOE2, for example, with a confidence level of 95%, α is 0.05, and *Z*-value is 1.96, with 5% MOE is the margin of error and p is the sample proportion. Because there was no previous study on COVID-19-related KAPP in Vietnam, the *p*-value will be assumed as 50% of residents had COVID-19 prevention practices. Therefore, the minimum required sample size was calculated as *N* = (1.96)^2^^*^0.5^*^0.5/(0.05)2 = 384, and if the assumption of a non-response rate was 10%, then the final minimum required sample size was 422.

With a convenient random sampling method, the study initially approached 1,257 responses, and then we continued to discard 294 subjects who did not complete all questions or who missed more than 50% of the questions. After cleaning the data, 963 eligible subjects equivalent to 76.6% of the given response rate were finalized to include in our study.

We used descriptive statistics to evaluate the sociodemographic characteristics of all participants, including age, gender, occupation, living location, religion, ethnicity, marital status, level of education, and source of information. We summarized the data using the mean and standard deviation (SD) or median and interquartile range (IQR) for continuous data, and frequencies and percentages for categorical data.

We used the ANOVA test to compare means between the three classifications of knowledge, attitude, perception, and practice (i.e., good, fair, and poor). We used the Chi-square test, Phi, and Crame's *V*-test to assess the difference according to qualitative variables. We used Pearson correlation analysis for four variables of KAPP with normal distribution. Some missing responses were assigned N/A in R software.

According to Bloom, B. S., the author defined the cutoff of 80% for each part based on the total expected score, the total scores for knowledge, attitude, and practice were categorized into good/positive or poor/negative (27, 28). In our study, we modified the categorization for KAPP as good for a score between 80 and 100%, fair for a score between 50 and 80%, and poor for a score of <50%. After assigning the knowledge, attitude, perception, and practice (KAPP) score to three levels, good, fair, and poor, we used the application of association rule mining in R software, also known as if–then rules {X -> Y}, to estimate the relationship probability of these levels among knowledge, attitude, perception, and practice. Based on the principle of association rule mining, which originated from machine learning, the symbol “{X -> Y}” does not imply a causal relationship between {X} and {Y}, it is merely an estimate of the conditional probability of {Y} given {X} (6, 29). The purpose of the three factors to measure in this rule (i.e., support, confidence, and lift) was to reduce the number of possible relationships (or possible rules) and selected only potentially “relevant” rules (29). We used “arules” and “arulesViz” packages to define potential relationships and plot the graph via the association rule method. As a result, numbered circles represented the generated rules, the size represented the rule's support, the color represented the rule's lift, and the arrow direction represented the rule's confidence when one moving toward the circles represented the antecedent or left-hand-side (LHS), in contrast, moving outwards the circle represented the consequent or right-hand-side (RHS) (29). The higher these values were, the more certain the probability of the relationship would be.

We set *a priori* level of significance at α = 0.05 for all statistical tests, and all tests were two-sided.

## Results

Data from a total of 963 eligible respondents were analyzed, and the scores and percentages (%) of the level consensus for knowledge, attitude, perception, and practice were shown in Table 1. In terms of evaluating the knowledge (K), our findings showed most participants (95.2%) reporting that the SARS-CoV-2 was transmitted through droplets by coughing, sneezing, or talking; 91.4% of participants had good knowledge about being able to contact the virus from daily activities such as touching a contaminated surface without washing their hands and then touching their eyes, nose, or mouth. However, 54.4% of people thought they could be infected by direct contact with the infected blood sample. The most common COVID-19 symptoms voted by the participants included fever or chills (94.5%), dry cough (88.9%), fatigue (87.2%), sore throat (86.5%), shortness of breath (84.9%), loss of smell (86.2%), and loss of taste (86.1%). The most voted high-risk factors for severe pneumonia or death from COVID-19 infection were being elderly, unvaccinated, obese, or having co-morbidities. Interestingly, about 56% of the opinions stated that asymptomatic patients would spread the virus to people around them.

Table 1Scores and percentages of the level consensus for knowledge, attitude, perception, and practice during the COVID-19 pandemic.

**Table:** 

**Knowledge**	**Total maximum score = 19**	**If response is no/i don't know = 0** **If response is yes (true) = +1; yes (false) = −1**
**Questions**	**Score of each question**	**Percentages (%)**
**Q1. In your opinion, which is the COVID-19 transmission route?**		
The virus can be transmitted through small particles that produce as an infected person coughs, sneezes, or talks.	+1	95.2 (910/956)
A person can get the virus when they touch a contaminated surface without washing their hands, and then touch their eyes, nose, or mouth.	+1	91.4 (874/956)
Contact directly with the infected blood sample may be a route of transmission of the COVID-19 virus	−1 (yes)	54.4 (521/957)
**Q2. Some symptoms of COVID-19 include:**		
Fever or chills (more common)	+1	94.5 (906/959)
Dry cough (more common)	+1	88.9 (853/959)
Fatigue (more common)	+1	87.2 (836/959)
Shortness of breath or difficulty breathing	+1	84.9 (814/959)
Sore throat	+1	86.5 (830/959)
Runny nose (Nasal discharge)	+1	66.6 (639/959)
Loss of smell (Anosmia)	+1	86.2 (827/959)
Loss of taste (Ageusia)	+1	86.1 (826/959)
Nausea, vomiting	+1	62.1 (596/959)
Headache	+1	66.8 (641/959)
Abdominal pain, diarrhea	+1	57.4 (550/959)
**Q3. In your opinion, which of the following subjects is at high risk of severe pneumonia or death when infected with COVID-19?**		
Old age	+1	94.5 (907/960)
Children	−1 (yes)	30.6(294/960)
Underlying medical conditions (cardiovascular disease, chronic kidney disease, diabetes mellitus,...)	+1	92.5 (888/960)
Obesity	+1	74 (710/960)
Underweight	−1 (yes)	47.1 (452/960)
Unvaccinated against the COVID-19	+1	83.5 (802/960)
Newly vaccinated with 1 dose of COVID-19 vaccine	−1 (yes)	51.2 (492/960)
Pregnant women	+1	35.4 (340/960)
**Q4. In your opinion, are the asymptomatic individuals infected with COVID-19 at risk of spreading the virus to people around them?**		
Yes	+1	55.8 (536/960)

**Table:** 

**Attitudes**	**Total maximum score** = **50**				
**Questions score of each item**	**Strongly disagree** +**1**	**Disagree** +**2**	**Neutral** +**3**	**Agree** +**4**	**Strongly agree** +**5**
**Q. Please indicate your level of agreement with the following statements about some measures to prevent COVID-19 infection (from 9K message issued by the**					
**Vietnamese Ministry of Health)**					
Keep your distance from others	11.2 (107/959)	2.2 (21/959)	15.6 (150/959)	28.7 (275/959)	42.3 (406/959)
Always wear a facemask in public places, crowded areas, medical care, or isolation areas	8.8 (85/961)	4.9 (47/961)	16.2 (156/961)	25.3 (243/961)	44.7 (430/961)
Do not gather in large groups	10.5 (101/961)	5.2 (50/961)	15.6 (150/961)	34.8 (334/961)	33.9 (326/961)
Always wash your hands with soap or hand sanitizer	6.4 (61/960)	6.3 (60/960)	13.3 (128/960)	33.8 (324/960)	40.3 (387/960)
Clean daily the high-touch surfaces and objects (doorknobs, phones, tablets, tables, chairs, etc.)	6.0 (58/959)	5.0 (48/959)	15.2 (146/959)	36.2 (347/959)	37.5 (360/959)
Keep the house clean, wash and keep the house with a good airflow	6.9 (66/958)	6.3 (60/958)	15.7 (150/958)	38.2 (366/958)	33.0 (316/958)
Border Protection: Strengthen patrols, control entry by land and sea road; strictly control, prevent and handle according to the law all illegal cross-border entry activities	5.9 (57/961)	6.5 (62/961)	16.3 (157/961)	35.9 (345/961)	35.4 (340/961)
Isolation sites safely: Strengthen surveillance, compliance check for mandatory isolated processes, and procedures and regulations in concentrated quarantine facilities.	5.9 (56/957)	6.9 (66/957)	14.1 (135/957)	39.6 (379/957)	33.5 (321/957)
People were not allowed to leave their homes unless absolutely necessary; If they need to leave home, they must strictly follow the epidemic prevention and control policies.	6.7 (64/960)	7.1 (68/960)	16.7 (160/960)	39.6 (380/960)	30.0 (288/960)
Public misinformation about COVID-19 caused confusion to the public's perception and impacts the effects of epidemic prevention and control from the Republican Party, the Government, and the People.	6.2 (60/962)	6.0 (58/962)	17.2 (165/962)	34.2 (329/962)	36.4 (350/962)

**Table:** 

**Perceptions**	**Total maximum score = 14**	**If response is no/i don't know = 0 if response is yes (true) = +1; yes (false)** = −**1**
**Questions**	**Score of each question**	**Percentages (%)**
**Q1. In your opinion, which of the following measures can help prevent the spread of COVID-19?**		
Avoid spending time in crowded areas	+1	75.3 (718/954)
Do not use public transportation	+1	63.8 (609/954)
Keep a distance of at least 2 m from each other	+1	75.9 (726/957)
Use a private bathroom if it's possible	+1	70.7 (672/951)
Restricting contact with pets and other animals or always wash your hands thoroughly with soap and water before and after handling animals	+1	67.2 (644/958)
Wear a mask when there are people around	+1	76.5 (726/949)
Cover your nose and mouth when coughing or sneezing	+1	76 (725/954)
Avoid touching your face (eyes, nose, and mouth)	+1	76.5 (727/950)
Always wash your hands	+1	76.4 (725/949)
Avoid sharing personal items with the others	+1	76.3 (726/951)
Clean daily the visibly dirty surfaces (such as phones, tablets, keyboards, remote controls, counters, countertops, bedside tables, doorknobs, bathroom fixtures, and toilets).	+1	80.1 (761/950)
**Q2. In your opinion, which of the following measures need to isolate at home for individuals who have been infected with COVID-19 (F0)?**		
Individuals positively infected with COVID-19 should use a separate bedroom and bathroom, avoid close contact with all family members, and stay at least 2 meters or 6 feet (about 2 arm lengths) apart from others.	+1	97.3 (935/961)
If a patient has to share space, make sure the room has good airflow (open the windows,...)	+1	82.1 (789/961)
Wear the facemask for both the sick and all family members.	+1	83.7 (804/961)
Just only wear a mask for the sick	−1 (yes)	25.2 (242/961) (yes)

**Table:** 

**Practices**	**Total maximum score = 11**	**If response is no/unknown = 0 if response is yes = +1**	
**Questions**	**Yes**	**No**	**Unknown**
**Q1. The following question examines what you have been doing in the past few days related to COVID-19 prevention. Please answer each issue in the question?**			
In recent days, have you been keeping your distance away from others?	71.6 (652/911)	21.2 (193/911)	7.2 (66/911)
In recent days, do you often wear a face mask when going to public places, crowded areas, and at medical facilities or isolation areas?	70.41 (652/926)	22.35 (207/926)	7.24 (67/926)
In recent days, have you restricted going to crowded places?	69.6 (641/921)	21.7 (200/921)	8.7 (80/921)
In recent days, have you limited communication with your neighbors?	69.1 (633/916)	21.4 (196/916)	9.5 (87/916)
In recent days, do you often wash your hands with soap or hand sanitizer?	80.61 (744/923)	10.18 (94/923)	9.21 (85/923)
In recent days, have you covered your nose and mouth when coughing or sneezing?	81.9 (750/916)	9.9 (91/916)	8.2 (75/916)
In recent days, have you avoided touching your face (eyes, nose, and mouth)?	74.7 (688/921)	15.1 (139/921)	10.2 (94/921)
In recent days, do you often clean high-touch surfaces and objects (doorknobs, phones, tablets, tables, chairs…)?	72.48 (669/923)	16.9 (156/923)	10.62 (98/923)
In recent days, do you often open the doors and windows, more frequently use the electric fans, and limit the use of air conditioners?	68.62 (632/921)	19.87 (183/921)	11.51 (106/921)
In recent days, have you followed not to leave your homes unless absolutely necessary; If you need to leave home, you must strictly follow the epidemic prevention and control policies.	74.73 (689/922)	14.86 (137/922)	10.41 (96/922)
Have you ever received two doses of the COVID-19 vaccine yet?	67.21 (615/915)	23.82 (218/915)	(82/915)

**Table:** 

**Q2. In recent days, what difficulties do you have when applying isolated measures due to COVID-19 pandemic (multiple answers)?**	**Percentages of choices (%)**
Approach hardly to medical services (medical examination or follow-up visits)	63.24 (609/963)
Income loss	51.71 (498/963)
Job loss	52.54 (506/963)
Not buying enough essential items (food, living items)	73.42 (707/963)
Purchase hardly some necessary medicine	58.67 (565/963)
Reduced income	63.03 (607/963)
Reduced mental health	65.42 (630/963)
Reduced physical health	56.18 (541/963)
Raised in a family conflict	31.78 (306/963)
Studying/working at home	64.8 (624/963)
**Q3. In recent days, what difficulties do you have when applying the preventive measures of COVID-19 pandemic (multiple answers)?**	
Applied strategies for the prevention of infections	57.32 (552/963)
Changing habits	65.42 (630/963)
Feeling unnecessary to apply infection prevention measures	41.95 (404/963)
Get uncomfortable when taking infection prevention measures	54.31 (523/963)
Shortage of personal protective equipment (facemask, hand sanitizer)	60.64 (584/963)
Without any difficulty	6.02 (58/963)

For the 9K message of attitude (A) evaluation, ~70% of residents presented with strong agreement about some measures to prevent COVID-19 infection, such as keeping some distance from others (71%); always wearing a face mask in public places, crowded areas, medical care, or isolation areas (70%); do not gather in large groups (68.7%); always wash hands with soap or hand sanitizer (74.1%); clean daily the high-touch surfaces and objects (doorknobs, phones, tablets, tables, chairs, etc.) (73.7%); keep the house clean, wash and keep the house with good airflow (71.2%); border protection (71.3%); and isolation sites safely (73.1%). Also, public misinformation about COVID-19 should be prohibited, according to 70.6% of the participants who voted.

For perception (P) evaluation, about 60–80% of the participants understood some measures to prevent the spread of COVID-19. In detail, 75.3% of participants reported that they should avoid spending time in crowded areas and 76.5% should wear a mask when there are people around, and avoid touching their faces (eyes, nose, and mouth). Roughly 76% of participants reported keeping a distance of at least 2 m, covering their nose and mouth when coughing or sneezing, washing hands, and avoiding sharing personal items with others; 80.1% of residents' responses recognized the prevention measure by cleaning daily visibly dirty surfaces (such as phones, tablets, keyboards, remote controls, counters, countertops, bedside tables, doorknobs, bathroom fixtures, and toilets). Regarding home isolation and quarantine guidance, more than 80% of people perceived the need to use a separate bedroom and bathroom (if possible) or to have good airflow if a patient had to share space, besides, avoiding close contact with other family members, staying away from others at least 2 m or 6 feet, and wearing facemask for both the sick and all family members. Still, 25.2% of people reported that the need to wear a face mask is for the sick only.

For practice (P) evaluation, for activities related to COVID-19 prevention in the past few days, around 80% of participants reported that they often washed their hands with soap or hand sanitizer while 82% of participants covered their nose and mouth when coughing or sneezing. Over 70% of people often wore face masks when going to public places, in crowded areas, at medical facilities or in isolation areas, kept their distance from others, avoided touching their face (eyes, nose, and mouth), cleaned high-touch surfaces and objects, and did not leave their homes unless necessary. In addition, 67.21% of respondents had already received two doses of the COVID-19 vaccine.

Regarding some difficulties that participants had when applying isolated measures. For example, there were 63.24% of participants who hardly approached medical services (i.e., medical examination or follow-up visits), 52.54% of them also experienced job loss, and more than 50% had income reduction or losses. In addition, there was an increase in family conflict (31.78% of participants), a decrease in mental health (65.42%), and a reduction in physical health (56.18%). Many participants stated that the lockdown forced them to work or study at home (64.8%) as well as not to prepare enough essential items (i.e., food and living items; 73.42%). In terms of some difficulties related to applying the preventive measures, many participants experienced changing habits (65.42%) and a shortage of personal protective equipment (i.e., face mask and hand sanitizer; 60.64%). Moreover, over 50% of participants felt difficult or uncomfortable when applying strategies to prevent infections, and ~42% of people thought it was unnecessary to follow these preventive measures. Only 6.02% of respondents reported having no difficulty.

Table 2 showed the characteristics of participants in the study according to the classification of knowledge, attitude, perception, and practice. Notably, there was enough statistical evidence to suggest that many variables correlated with the KAPP score. This included variables such as age, medical staff group, living accommodation, religious belief, marital status, education level, occupation, and information access (i.e., television, newspaper, the Vietnamese Ministry of Health website, Ho Chi Minh CDC website, and friends). In contrast, while gender, location, and ethnic group were statistically related to knowledge, perception, and practice score, there was enough evidence to suggest that they were not related to attitudes (*p* = 0.96, 0.61, and 0.077, respectively). Interestingly, local radio was related to only practice scores.

**Table 2:** Characteristics of the subjects participating in the study according to the classification of knowledge, attitudes, perceptions, and practices.

**Characteristics**	**Knowledge**				**Attitudes**				**Perceptions**				**Practices**			
	**Good**	**Fair**	**Poor**	***p*** **-value**	**Good**	**Fair**	**Poor**	***p*** **-value**	**Good**	**Fair**	**Poor**	***p*** **-value**	**Good**	**Fair**	**Poor**	***p*** -value^*^
	***N*** = **210**	***N*** = **611**	***N*** = **139**		***N*** = **521**	***N*** = **386**	***N*** = **55**		***N*** = **552**	***N*** = **222**	***N*** = **187**		***N*** = **542**	***N*** = **136**	***N*** = **214**	
**Age (years)**	29.98 (8.90)	45.97 (16.48)	45.65 (13.79)	<0.001^*^	35.65 (14.35)	51.60 (13.69)	41.50 (16.16)	<0.001^*^	36.26 (14.23)	46.81 (15.21)	55.21 (13.04)	<0.001^*^	36.27 (14.05)	46.36 (15.60)	53.66 (13.76)	<0.001^*^
**Gender**				<0.001^*^				0.973				0.001^*^				0.002^*^
Male	83 (39.52%)	190 (31.30%)	66 (48.18%)		185 (35.71%)	136 (35.42%)	20 (37.04%)		218 (39.78%)	75 (33.94%)	47 (25.27%)		215 (39.89%)	49 (36.57%)	56 (26.29%)	
Female	127 (60.48%)	417 (68.70%)	71 (51.82%)		333 (64.29%)	248 (64.58%)	34 (62.96%)		330 (60.22%)	146 (66.06%)	139 (74.73%)		324 (60.11%)	85 (63.43%)	157 (73.71%)	
**Medical staff**				<0.001^*^				<0.001^*^				<0.001^*^				<0.001^*^
Yes	105 (50.00%)	76 (12.46%)	5 (3.60%)		151 (28.98%)	25 (6.49%)	10 (18.18%)		148 (26.81%)	31 (13.96%)	7 (3.76%)		152 (28.04%)	12 (8.82%)	13 (6.10%)	
No	105 (50.00%)	534 (87.54%)	134 (96.40%)		370 (71.02%)	360 (93.51%)	45 (81.82%)		404 (73.19%)	191 (86.04%)	179 (96.24%)		390 (71.96%)	124 (91.18%)	200 (93.90%)	
**Location**				<0.001^*^				0.611				0.003^*^				0.002^*^
Live in the inner Ho Chi Minh city	152 (72.38%)	473 (77.41%)	46 (33.09%)		368 (70.63%)	269 (69.69%)	35 (63.64%)		360 (65.22%)	166 (74.77%)	146 (78.07%)		368 (67.90%)	83 (61.03%)	173 (80.84%)	
Live in suburban in Ho Chi Minh city	31 (14.76%)	64 (10.47%)	47 (33.81%)		72 (13.82%)	63 (16.32%)	7 (12.73%)		101 (18.30%)	28 (12.61%)	13 (6.95%)		93 (17.16%)	25 (18.38%)	16 (7.48%)	
Thu Duc city	13 (6.19%)	35 (5.73%)	27 (19.42%)		37 (7.10%)	32 (8.29%)	6 (10.91%)		49 (8.88%)	14 (6.31%)	12 (6.42%)		43 (7.93%)	13 (9.56%)	12 (5.61%)	
Others	14 (6.67%)	39 (6.38%)	19 (13.67%)		44 (8.45%)	22 (5.70%)	7 (12.73%)		42 (7.61%)	14 (6.31%)	16 (8.56%)		38 (7.01%)	15 (11.03%)	13 (6.07%)	
**Living**				<0.001^*^				<0.001^*^				0.001^*^				0.006^*^
**accommodation**																
Private house	146 (71.22%)	390 (63.93%)	48 (34.78%)		346 (67.32%)	204 (52.85%)	36 (65.45%)		359 (65.87%)	113 (50.90%)	113 (60.43%)		347 (64.74%)	72 (52.94%)	117 (54.67%)	
Motel room	59 (28.78%)	220 (36.07%)	90 (65.22%)		168 (32.68%)	182 (47.15%)	19 (34.55%)		186 (34.13%)	109 (49.10%)	74 (39.57%)		189 (35.26%)	64 (47.06%)	97 (45.33%)	
**Ethnic group**				0.002^*^				0.077				0.015^*^				0.004^*^
Kinh	196 (94.23%)	584 (96.85%)	112 (90.32%)		483 (93.97%)	358 (97.02%)	53 (98.15%)		509 (93.39%)	204 (98.08%)	180 (98.36%)		500 (93.81%)	119 (92.97%)	205 (99.51%)	
Hoa	8 (3.85%)	15 (2.49%)	12 (9.68%)		25 (4.86%)	10 (2.71%)	0 (0.00%)		29 (5.32%)	3 (1.44%)	3 (1.64%)		26 (4.88%)	8 (6.25%)	1 (0.49%)	
Others	4 (1.92%)	4 (0.66%)	0 (0.00%)		6 (1.17%)	1 (0.27%)	1 (1.85%)		7 (1.28%)	1 (0.48%)	0 (0.00%)		7 (1.31%)	1 (0.78%)	0 (0.00%)	
**Religions**				<0.001^*^				<0.001^*^				<0.001^*^				<0.001^*^
No religion	132 (63.77%)	156 (25.66%)	34 (25.19%)		250 (48.36%)	54 (14.21%)	19 (34.55%)		258 (47.25%)	56 (25.34%)	8 (4.35%)		256 (47.67%)	33 (25.00%)	14 (6.57%)	
Religious people	75 (36.23%)	452 (74.34%)	101 (74.81%)		267 (51.64%)	326 (85.79%)	36 (65.45%)		288 (52.75%)	165 (74.66%)	176 (95.65%)		281 (52.33%)	99 (75.00%)	199 (93.43%)	
**Marriage_status**				<0.001^*^				<0.001^*^				<0.001^*^				<0.001^*^
Single	127 (61.35%)	210 (34.71%)	50 (36.50%)		256 (49.90%)	112 (29.17%)	18 (33.33%)		270 (49.45%)	72 (33.03%)	45 (24.19%)		262 (49.06%)	53 (39.26%)	51 (24.06%)	
Married	78 (37.68%)	289 (47.77%)	56 (40.88%)		214 (41.72%)	179 (46.61%)	33 (61.11%)		232 (42.49%)	113 (51.83%)	79 (42.47%)		235 (44.01%)	57 (42.22%)	96 (45.28%)	
Widow wife/husband	0 (0.00%)	6 (0.99%)	5 (3.65%)		5 (0.97%)	6 (1.56%)	0 (0.00%)		5 (0.92%)	5 (2.29%)	1 (0.54%)		4 (0.75%)	3 (2.22%)	4 (1.89%)	
Divorce/separated	2 (0.97%)	100 (16.53%)	26 (18.98%)		38 (7.41%)	87 (22.66%)	3 (5.56%)		39 (7.14%)	28 (12.84%)	61 (32.80%)		33 (6.18%)	22 (16.30%)	61 (28.77%)	
**Education**				<0.001^*^				<0.001^*^				<0.001^*^				<0.001^*^
Elementary/middle/high school	27 (12.86%)	410 (67.10%)	120 (86.33%)		184 (35.32%)	346 (89.64%)	27 (49.09%)		202 (36.59%)	172 (77.48%)	183 (97.86%)		193 (35.61%)	107 (78.68%)	210 (98.13%)	
Vocational training	12 (5.71%)	17 (2.78%)	10 (7.19%)		25 (4.80%)	13 (3.37%)	1 (1.82%)		26 (4.71%)	11 (4.95%)	2 (1.07%)		30 (5.54%)	5 (3.68%)	2 (0.93%)	
University	149 (70.95%)	157 (25.70%)	8 (5.76%)		270 (51.82%)	23 (5.96%)	22 (40.00%)		279 (50.54%)	34 (15.32%)	2 (1.07%)		271 (50.00%)	24 (17.65%)	2 (0.93%)	
Post-graduation	22 (10.48%)	27 (4.42%)	1 (0.72%)		42 (8.06%)	4 (1.04%)	5 (9.09%)		45 (8.15%)	5 (2.25%)	0 (0.00%)		48 (8.86%)	0 (0.00%)	0 (0.00%)	
**Occupation**				<0.001^*^				<0.001^*^				<0.001^*^				<0.001^*^
Full time	117 (57.35%)	176 (29.04%)	21 (15.11%)		227 (44.42%)	66 (17.10%)	24 (44.44%)		253 (46.68%)	44 (19.91%)	18 (9.63%)		249 (46.89%)	27 (19.85%)	20 (9.35%)	
Part time	61 (29.90%)	127 (20.96%)	37 (26.62%)		139 (27.20%)	75 (19.43%)	10 (18.52%)		143 (26.38%)	43 (19.46%)	39 (20.86%)		141 (26.55%)	32 (23.53%)	38 (17.76%)	
Unemployment	22 (10.78%)	293 (48.35%)	72 (51.80%)		131 (25.64%)	239 (61.92%)	17 (31.48%)		132 (24.35%)	127 (57.47%)	128 (68.45%)		128 (24.11%)	74 (54.41%)	152 (71.03%)	
Retirement	4 (1.96%)	10 (1.65%)	9 (6.47%)		14 (2.74%)	6 (1.55%)	3 (5.56%)		14 (2.58%)	7 (3.17%)	2 (1.07%)		13 (2.45%)	3 (2.21%)	4 (1.87%)	
**Television**				<0.001^*^				<0.001^*^				<0.001^*^				<0.001^*^
Yes	104 (49.52%)	139 (22.75%)	44 (31.65%)		211 (40.50%)	56 (14.51%)	21 (38.18%)		238 (43.12%)	41 (18.47%)	9 (4.81%)		228 (42.07%)	31 (22.79%)	13 (6.07%)	
No	106 (50.48%)	472 (77.25%)	95 (68.35%)		310 (59.50%)	330 (85.49%)	34 (61.82%)		314 (56.88%)	181 (81.53%)	178 (95.19%)		314 (57.93%)	105 (77.21%)	201 (93.93%)	
**Newspaper**				<0.001^*^				<0.001^*^				<0.001^*^				<0.001^*^
Yes	32 (15.24%)	33 (5.40%)	4 (2.88%)		56 (10.75%)	9 (2.33%)	3 (5.45%)		57 (10.33%)	9 (4.05%)	3 (1.60%)		59 (10.89%)	3 (2.21%)	3 (1.40%)	
No	178 (84.76%)	578 (94.60%)	135 (97.12%)		465 (89.25%)	377 (97.67%)	52 (94.55%)		495 (89.67%)	213 (95.95%)	184 (98.40%)		483 (89.11%)	133 (97.79%)	211 (98.60%)	
**Radio**				0.464				0.199				0.086				0.013^*^
Yes	8 (3.81%)	17 (2.78%)	2 (1.44%)		19 (3.65%)	8 (2.07%)	0 (0.00%)		21 (3.80%)	4 (1.80%)	2 (1.07%)		23 (4.24%)	1 (0.74%)	2 (0.93%)	
No	202 (96.19%)	594 (97.22%)	137 (98.56%)		502 (96.35%)	378 (97.93%)	55 (100.00%)		531 (96.20%)	218 (98.20%)	185 (98.93%)		519 (95.76%)	135 (99.26%)	212 (99.07%)	
**Internet**				0.067				0.535				0.859				0.308
Yes	204 (97.14%)	591 (96.73%)	129 (92.81%)		500 (95.97%)	374 (96.89%)	52 (94.55%)		530 (96.01%)	215 (96.85%)	180 (96.26%)		522 (96.31%)	130 (95.59%)	210 (98.13%)	
No	6 (2.86%)	20 (3.27%)	10 (7.19%)		21 (4.03%)	12 (3.11%)	3 (5.45%)		22 (3.99%)	7 (3.15%)	7 (3.74%)		20 (3.69%)	6 (4.41%)	4 (1.87%)	
**Website_**				<0.001^*^				<0.001^*^				<0.001^*^				<0.001^*^
**of_ministry**																
Yes	130 (61.90%)	144 (23.57%)	24 (17.27%)		246 (47.22%)	33 (8.55%)	19 (34.55%)		259 (46.92%)	31 (13.96%)	8 (4.28%)		254 (46.86%)	19 (13.97%)	11 (5.14%)	
No	80 (38.10%)	467 (76.43%)	115 (82.73%)		275 (52.78%)	353 (91.45%)	36 (65.45%)		293 (53.08%)	191 (86.04%)	179 (95.72%)		288 (53.14%)	117 (86.03%)	203 (94.86%)	
**HCDC_website**				<0.001^*^				<0.001^*^				<0.001^*^				<0.001^*^
Yes	108 (51.43%)	106 (17.35%)	42 (30.22%)		182 (34.93%)	61 (15.80%)	12 (21.82%)		216 (39.13%)	29 (13.06%)	11 (5.88%)		205 (37.82%)	23 (16.91%)	17 (7.94%)	
No	102 (48.57%)	505 (82.65%)	97 (69.78%)		339 (65.07%)	325 (84.20%)	43 (78.18%)		336 (60.87%)	193 (86.94%)	176 (94.12%)		337 (62.18%)	113 (83.09%)	197 (92.06%)	
**Friends**				<0.001^*^				<0.001^*^				<0.001^*^				<0.001^*^
Yes	143 (68.10%)	507 (82.98%)	100 (71.94%)		376 (72.17%)	337 (87.31%)	37 (67.27%)		388 (70.29%)	188 (84.68%)	174 (93.05%)		381 (70.30%)	110 (80.88%)	206 (96.26%)	
No	67 (31.90%)	104 (17.02%)	39 (28.06%)		145 (27.83%)	49 (12.69%)	18 (32.73%)		164 (29.71%)	34 (15.32%)	13 (6.95%)		161 (29.70%)	26 (19.12%)	8 (3.74%)	

* The statistical significant tests used include chi-square, Phi, and Cramer's V for qualitative variables and ANOVA test for quantitative variables.

**Figure 1:**
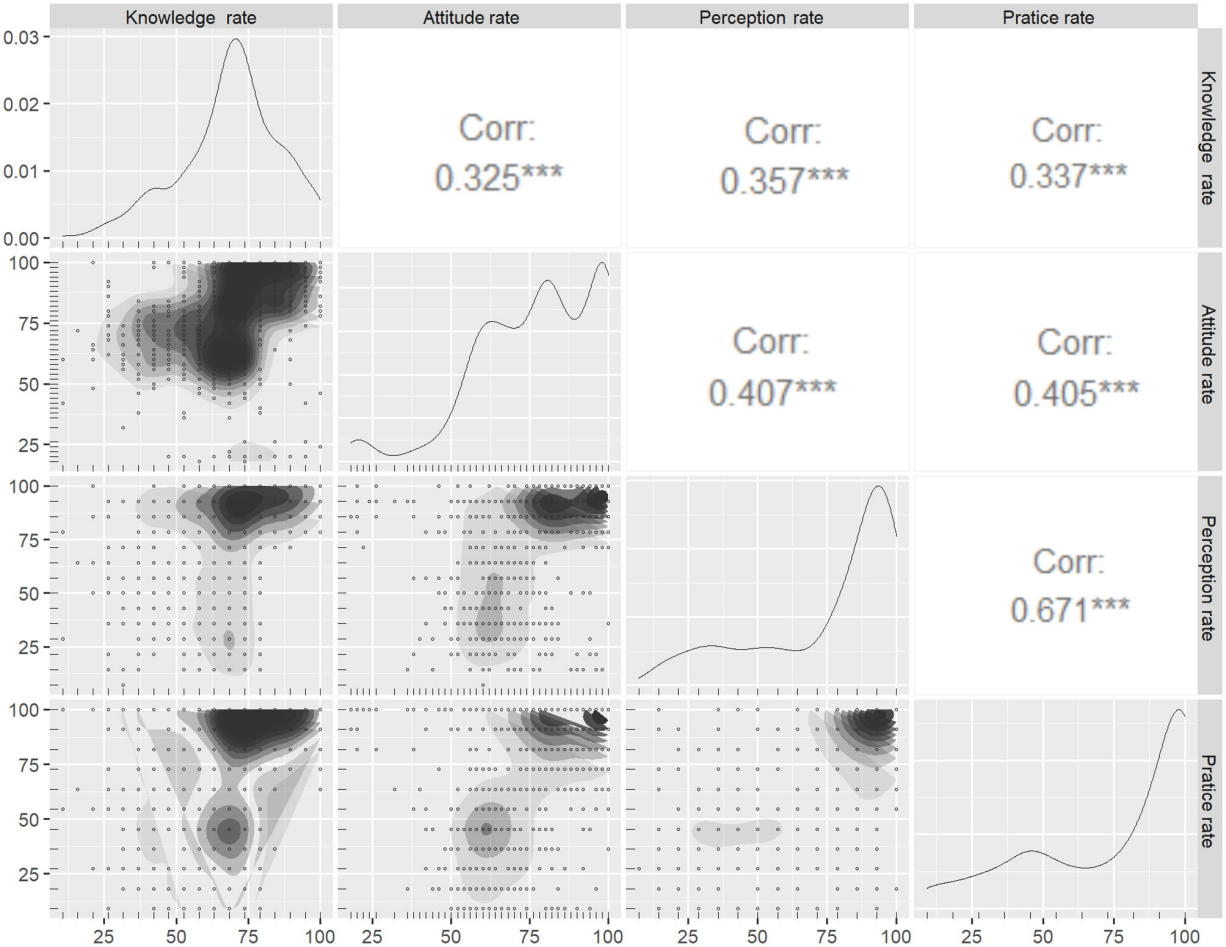
Pearson correlation among knowledge, attitudes, perceptions, and practices. ^***^P-value < 0.001.

**Figure 2:**
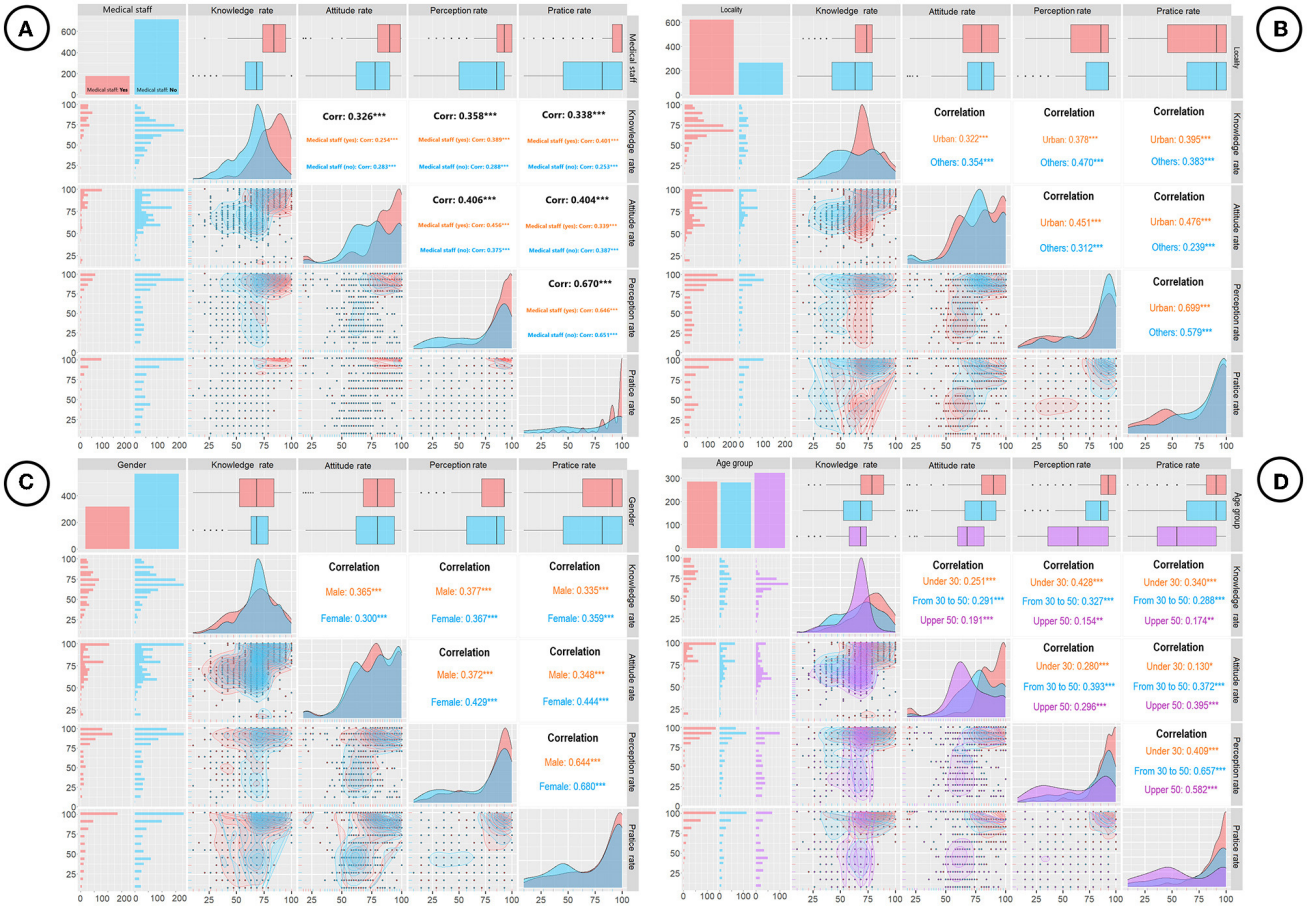
**(A)** Pearson correlation of knowledge, attitudes, perceptions, and practices (KAPP) in terms of occupation (medical staff). **(B)** Pearson correlation of KAPP with locality. **(C)** Pearson correlation of KAPP with gender. **(D)** Pearson correlation of KAPP with age group. ^*^P < 0.05, ^**^P < 0.01, ^***^P < 0.001.

The overall K, A, P, and P scores were 68.67% ± 17.16, 77.33% ± 18.71, 74.7% ± 26.25, and 72.31% ± 31, respectively. The medical staff group's mean KAPP score (%) was mostly higher than those scored in the non-medical staff group. The mean ± SD of the knowledge score, attitude score, perception score, and practice score (%) were 81.48 ± 15.38, 84.17 ± 20.16, 87.43 ± 16.8, and 84.2 ± 27.03 in the medical group, respectively, whereas those in the non-medical group were 65.31 ± 16.6, 75.6 ± 18.17, 71.44 ± 27.4, and 66.04 ± 33.8, respectively. Because the KAPP score is followed by a normal distribution, we performed Pearson correlation to measure the strength of the linear correlation between the two scores. The results in Figure 1 showed positive, medium–strong, and significant correlations between knowledge and practice (correlation coefficient *r* = 0.337), attitude and practice (*r* = 0.405), and perception and practice (*r* = 0.671; *p* < 0.05). We also performed sub-group correlation analysis in terms of occupation (medical staff), locality, gender, and age group with KAPP scores (Figure 2). There were mostly medium–strong and significant correlations of each sub-group with KAPP score (i.e., medical staff–non-medical staff, urban–non-urban, male–female, and under 30–from 30 to 50–upper 50; *p* < 0.05).

**Figure 3:**
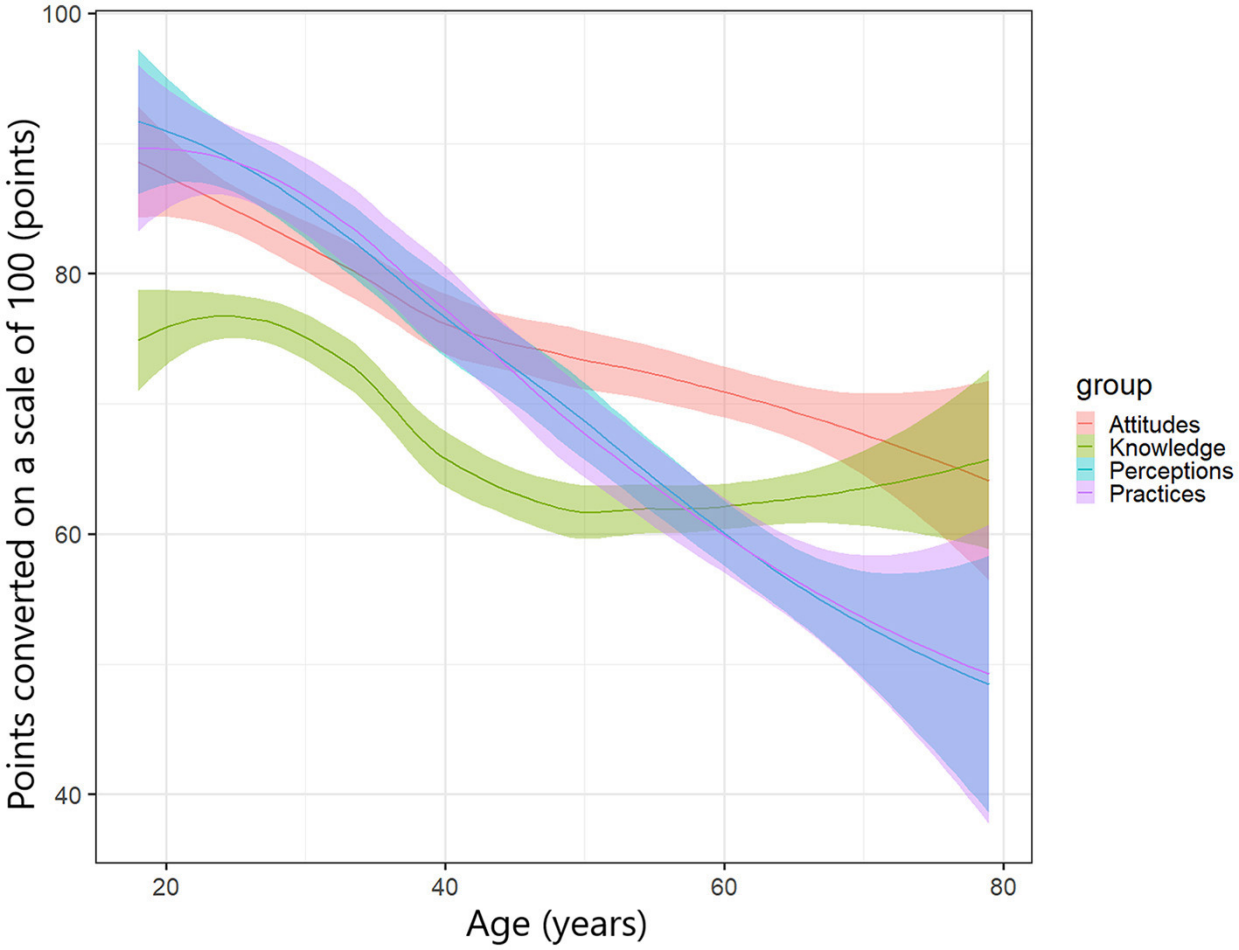
The relationship among knowledge, attitudes, perceptions, and practices followed by age.

**Figure 4:**
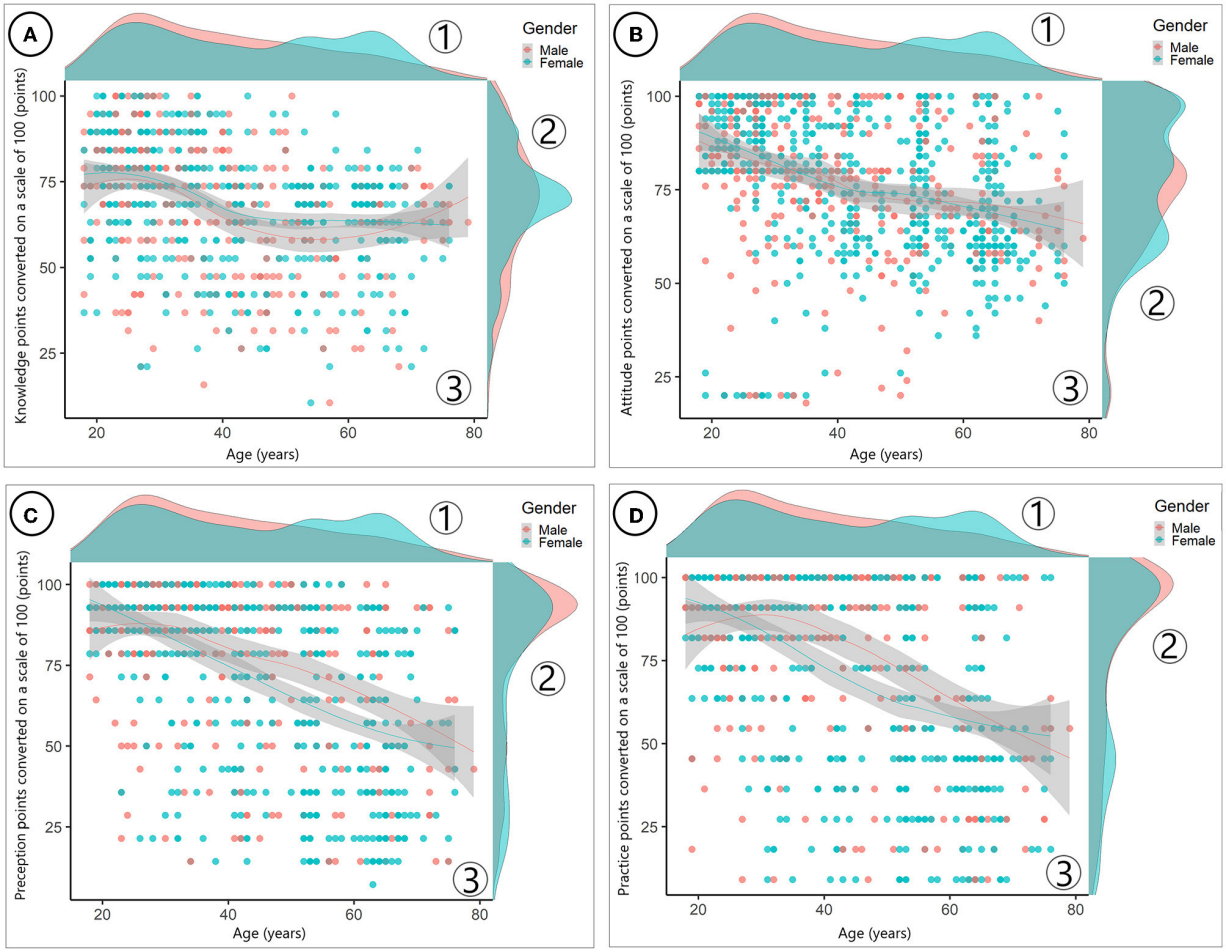
**(A)** The relationship between knowledge with age and gender. **(B)** The relationship between attitudes with age and gender. **(C)** The relationship between perceptions with age and gender. **(D)** The relationship between practices with age and gender.

Figure 3 shows the relationship among knowledge, attitude, perception, and practice followed by age. We demonstrated earlier from Table 2 that participants' age was significantly related to all scores of KAPP. In general, attitude, perception, and practice gradually decreased over time when individuals were 30 years or older; while knowledge first increased for those people below 30, after that it gradually decreased within the age range 30 to 50 years, then turned back to slowly increase at aged 50 and over, especially in those 60 s and 80 s. When we analyzed more deeply with both age and gender in Figure 4, the previous tendency had changed a little bit for distinct male and female participants, especially in knowledge, perception, and practice. This trend appeared to make a slight increase in male compared to female participants due to the different scores of levels, highly in 60–80 ages in knowledge and 20–40 ages in perception and practice.

**Figure 5:**
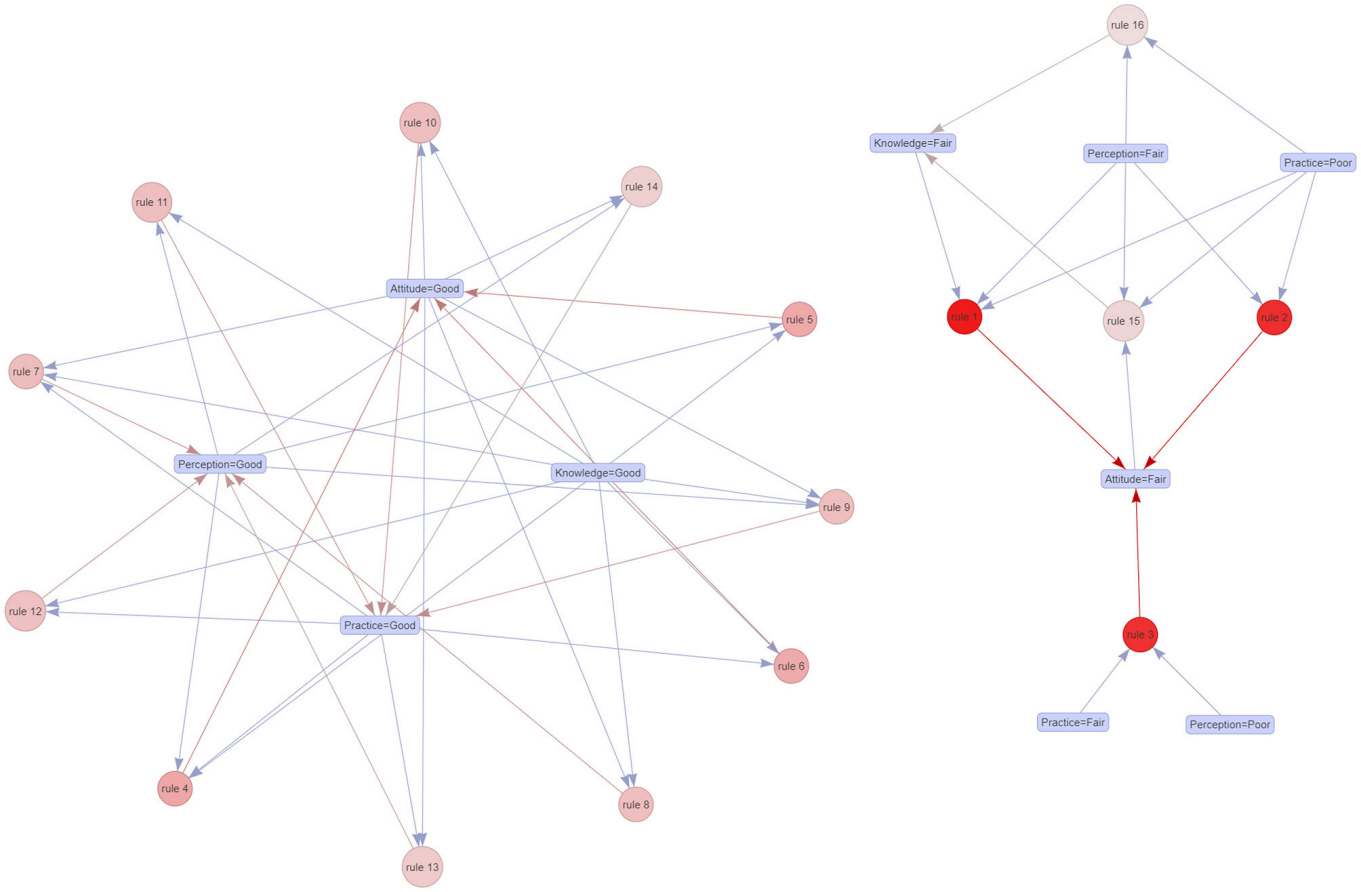
Association rules among knowledge, attitudes, perceptions, and practices.

A detailed relationship of KAPP scores through the association rules method was shown in Figure 5 and Table 3. We found 16 rules to estimate the conditional probability of {Y} given {X} or right-hand-side (rhs) given by left-hand-side (lhs). All of the lift values in Table 3 were >1 means all items of KAPP classifications of both rhs and lhs were likely to be presented together. All of the items in these rules that were involved would appear with the probability given by the rule's confidence from 0.86 to 0.94 (or 86–94%) with a support of 3–39%. We could observe that rule 9 stated that {Knowledge=Good, Attitude=Good, Perception=Good -> Practice=Good} had the support of 17.6% and a confidence of 94%, and rules 4 and 7 stated that {Knowledge=Good, Perception=Good, Practice=Good -> Attitude=Good} and {Knowledge=Good, Attitude=Good, Practice=Good -> Perception=Good}, respectively, had the support of 17.6% and a confidence of 91.8%. The plotting in Figure 5 notably showed that the circles with darker colors of rule 1, rule 2, and rule 3 told us the high rule's lifts (>2). These rules had shown ~88–90% of the times that the participants had levels of {Knowledge=Fair, Perception=Fair, Practice=Poor} given associated with {Attitude=Fair} as well. The same trend was also observed over rules 15 and 16 where ~86–89% of the time the participants had levels of {Attitude=Fair, Perception=Fair, Practice=Poor} given associated with {Knowledge=Fair}.

**Table 3:** The index evaluates the relationship between knowledge, attitudes, perceptions, and practices through the association rules method.

**Rule**	**Lhs {X}**		**Rhs {Y}**	**Support**	**Confidence**	**Coverage**	**Lift**	**Count**
(1)	{Knowledge=Fair, **Perception=Fair, Practice=Poor}**	=>	**{Attitude=Fair}**	**0.071**	**0.9**	0.079	2.23	63
(2)	**{Perception=Fair, Practice=Poor}**	=>	{**Attitude=Fair**}	**0.08**	**0.877**	0.091	2.172	71
(3)	{Perception=Poor, Practice=Fair}	=>	{Attitude=Fair}	0.03	0.871	0.035	2.158	27
(4)	{Knowledge=Good, Perception=Good, Practice=Good}	=>	{Attitude=Good}	0.176	0.918	0.192	1.699	156
(5)	{Knowledge=Good, Perception=Good}	=>	{Attitude=Good}	0.187	0.917	0.204	1.698	166
(6)	{Knowledge=Good, Practice=Good}	=>	{Attitude=Good}	0.192	0.909	0.211	1.683	170
(7)	{Knowledge=Good, Attitude=Good, Practice=Good}	=>	{Perception=Good}	0.176	0.918	0.192	1.559	156
(8)	{Knowledge=Good, Attitude=Good}	=>	{Perception=Good}	0.187	0.917	0.204	1.558	166
(12)	{Knowledge=Good, Practice=Good}	=>	{Perception=Good}	0.192	0.909	0.211	1.545	170
(13)	{Attitude=Good, Practice=Good}	=>	{Perception=Good}	0.391	0.868	0.451	1.474	347
(9)	**{Knowledge=Good, Attitude=Good, Perception=Good}**	=>	**{Practice=Good}**	**0.176**	**0.94**	0.187	1.552	156
(10)	{Knowledge=Good, Attitude=Good}	=>	{Practice=Good}	0.192	0.939	0.204	1.551	170
(11)	{Knowledge=Good, Perception=Good}	=>	{Practice=Good}	0.192	0.939	0.204	1.551	170
(14)	{Attitude=Good, Perception=Good}	=>	{Practice=Good}	0.391	0.876	0.446	1.447	347
(15)	{Attitude=Fair,**Perception=Fair, Practice=Poor}**	=>	{**Knowledge=Fair}**	**0.071**	**0.887**	0.08	1.41	63
(16)	**{Perception=Fair, Practice=Poor}**	=>	**{Knowledge=Fair}**	**0.079**	**0.864**	0.091	1.374	70

The bold values indicate the relationship {Practice=Good} and {Practice=Poor} with Knowledge, Attitude, Perception.

## Discussion

This is the latest cross-sectional survey-based study with 963 participants in Vietnam, which contributed to a better understanding of the gap in knowledge, attitude, perception, and practice among residents in controlling the COVID-19 pandemic. Regarding 21 evaluated questions, the study provided valuable insights into the residents' KAPP scores which were 68.67% ± 17.16, 77.33% ± 18.71, 74.7% ± 26.25, and 72.31% ± 31, respectively.

### KAPP scores

Our results were relatively lower than the China population, with the knowledge, attitude, and practice (KAP) scoring rates being 85.2, 92.9, and 84.4%, respectively, but higher than the survey in the Northeast Ethiopia population (knowledge and practice scores were 33.9 and 47.3%, respectively) (7, 30, 31). However, the study in Northeast Ethiopia was conducted among chronic disease patients, which could have affected their perception to evaluate the score, while the study in China was conducted among residents.

In our population, respondents reported that their practices in wearing a face mask, washing hands or using hand sanitizer, and maintaining social distancing were 70.41, 80.61, and 74.7%, respectively. The results were lower than those in the North Central Nigeria survey (82.3, 96.4, and 92.7%, respectively) (32). This could be partly explained by the difference in population age surveyed between the two studies. The study in North Central Nigeria was mainly conducted in the young group aged 18–39 years (80.6%); compared to our research, only 49.74% of participants were under 40 years.

In comparison with a survey in Malaysia, avoiding crowds or limited communication with neighbors were lower in our study (69.1 vs. 83.4%). The satisfaction of wearing a face mask was approximately similar, with over 80%, between these two populations. However, there is a dramatic difference in the pleasure of using hand sanitizer, which was 81.9% in our study vs. only 51.2% in the Malaysian survey (33). This might reflect the government policy and the health education programs during the pandemic.

Another study from Japan, which included 79.0% of undergraduate students and 83.7% of Japanese residents, revealed that 100% of respondents retained knowledge on avoiding confined spaces, crowded places, and close contact circumstances. The study also showed that living in capital areas with high fundamental understanding and accurate information donated positively to preventive activities (34). Compared to our study, 75.3% of participants understood that they should avoid spending time in crowded areas, 76% of participants reported keeping a distance of at least 2 m, and more than 80% of people perceived the need to avoid close contact with other family members if you were the sick.

### Some difficulties when applying isolated measures and preventive measures

In late April 2021, Ho Chi Minh City quickly became an epicenter of the outbreak over a few months. Moreover, the emergence of the SARS-CoV-2 Omicron variant has created great global distress (35). The resident's life was severely influenced during this period. Our study found that residents hardly approached medical services (63.24%), had massive job losses (52.54%), and reduced or lost income (52–63%). Moreover, this strict social isolation also increased family conflict (31.78%) and decreased physical and mental health (56–65%). As same as our report, only 37.6% of Egyptians assumed their salary would be continued if they were in isolation which reflected the financial worries of residents in periods of social isolation (36).

A study across 63 countries revealed many factors attributed to stress during the COVID-19 pandemic, including female sex, unmarried status, inadequate education level, obligatory quarantine, and uncomfortable feelings during isolation (37). Another global survey concluded that less-educated individuals experienced a higher chance of developing post-traumatic stress disorder (PTSD) symptoms as opposed to those with university educational levels (38). There was one study to evaluate the fear level related to COVID-19, and the results showed three out of 10 (30.5%) people responded positively about fear (39). Therefore, assessing residents' difficulties when applying preventive measures under national quarantine was necessary to prevent and control the COVID-19 pandemic comprehensively.

### Sociodemographic characteristics with KAPP scores

A survey among Syrian residents indicated that low knowledge scores were significantly associated with low education levels (*P* < 0.05). In addition, poor preventive practices were common among young, male sex, and unemployed participants (*P* < 0.01) (40). While the results from the Egyptian population showed the knowledge score was particularly inferior among older, lower levels of education, lower-income people, and rural inhabitants, knowledge was gained mainly through social media (66.9%) and the internet (58.3%) (36). A Bangladesh survey showed that practice factors were associated with female sex, older age, higher education, employment, high monthly family income, urban area residence, and positive attitudes (41). These results corresponded well with our study where the practice had significantly related to almost all factors such as age, gender, education level, occupation, healthcare website, and radio. Furthermore, the Shanghai population acquired knowledge and skills mostly through the Internet and television (42).

### KAPP scores in medical staff

The KAPP scores of the medical personnel in our sample were 81.48 ± 15.38, 84.17 ± 20.16, 87.43 ± 16.8, and 84.2 ± 27.03 respectively, which were higher than the non-medical group (65.31 ± 16.6, 75.6 ± 18.17, 71.44 ± 27.4, and 66.04 ± 33.8, respectively). A study from China recently assessed undergraduate students, in which the levels of knowledge from public universities and medical majors were significantly higher than those from private academics and non-medical fields (*p* < 0.05) (43). One study in Vietnam performed on healthcare workers (HCWs) indicated that HCWs had good knowledge (91.3%), a positive attitude (71.5%), and appropriate practice (83.1%) toward COVID-19 prevention (44). In addition, a study in China also showed that 89% of HCWs had adequate knowledge and 89.7% of HCWs followed proper practices (45). Therefore, health instruction programs aimed at enhancing COVID-19 understanding help maintain good attitudes and suitable practices.

### The conditional probability of all scores of KAPP

KAPP score was the most important factor in the principles of health behavior models. Our study showed positive, medium–strong correlations between knowledge and practice (*r* = 0.337), attitude and practice (*r* = 0.405), and perception and practice (*r* = 0.671; *p* < 0.05). This showed higher results compared with the results from Syrian residents of knowledge-practice and attitude-practice (i.e., 0.198 and 0.210, respectively) (40). Furthermore, applying the association rules method for the KAPP score, we found 16 rules to describe the conditional probabilities among KAPP scores. Mainly, 94% confident probability of participants had {Knowledge=Good, Attitude=Good, Perception=Good}, and they would have {Practice=Good} as well (in rule 9 with support of 17.6%). In opposition to ~86–90% probability of participants having either {Attitude=Fair} or {Knowledge=Fair}, they would have {Perception=Fair, Practice=Poor} (according to rules 1, 2, 15, and 16 with a support of 7–8%). The results affirmed the good internal relationship of KAPP scores and created a hierarchy of healthcare educational goals.

The positive results from the research on citizens' knowledge, attitude, and behavior during the COVID-19 pandemic have significant implications for practice. The study demonstrated that citizens have a good understanding of the issues related to the pandemic, and this could be leveraged to develop more effective and targeted interventions. In particular, the study's findings suggest that a focus on improving the attitudes and behaviors of citizens could lead to a more positive impact on the pandemic response. Such interventions could include public awareness campaigns, educational programs, or policies encouraging positive attitudes and behaviors such as wearing masks, maintaining social distancing, and getting vaccinated. Policymakers can also use the study's results to develop more effective policies that can improve citizens' overall health and wellbeing during pandemics. Overall, the findings of this study can be used to inform and guide the development of targeted interventions aimed at improving the knowledge, attitudes, and behaviors of citizens, leading to better health outcomes for both individuals and society as a whole in the face of pandemics such as COVID-19 or any future public health crisis.

### Strengths and limitations

Our study was the latest survey in Vietnam during the longest and the most severe city-wide lockdown due to the COVID-19 pandemic. Based on that, there is a comprehensive understanding and assessment of the public's cognition and health behavior through KAPP scores. Other than that, we also compared the KAPP scores between medical staff and non-medical staff sub-groups and investigated some recent difficulties while applying measures throughout the outbreak, for which there were no previous studies performed with full appreciation of health behavior regarding COVID-19-related prevention practices among residents.

There were also some limitations in our study. First, our study did not include data on residents who refused to enroll or did not finish all the questions items. Second, KAPP scores might not reflect perfectly the COVID-19 understanding of all populations in all regions in Vietnam because this study was carried out during the rapid rise period of the COVID-19 outbreak in Ho Chi Minh City. Finally, some special groups may be ignored through the online nature of the survey.

## Conclusion

In addition to the government's directives and policies, citizen's knowledge, attitude, perception, and practice are considered the critical preventive measures during the COVID-19 pandemic. Educated good knowledge, good attitude, and good perception of residents should also be paid more attention because they would be associated with good practice in health behavior regarding COVID-19-related prevention practice among residents.

## Data availability statement

The original contributions presented in the study are included in the article/Supplementary material, further inquiries can be directed to the corresponding authors.

## Ethics statement

The study obtained academic and ethical approval from the Institutional Review Board Office of the School of Tropical Medicine and Global Health, Nagasaki University, Japan (Reference number: NU_TMGH_2020_118_1). The patients/participants provided their written informed consent to participate in this study.

## Author contributions

NH and TTL conceived the study. NH, TTL, TL, and ML were principally responsible for formulating the study design and acquisition of data. TTL, TL, ML, NT, and TV made substantial contributions to building the questionnaire. TL and LV were principally responsible for the analysis and interpretation of data. TTL, TL, and AM drafted the manuscript. All authors revised the manuscript critically for important intellectual content, formulated the tables, adjusted the changes required for the submission, and read and approved the final version of the manuscript.
