# Supplementary material for: Knowledge, attitude, and perception regarding COVID-19-related prevention practice among residents in Vietnam: a cross-sectional study
Source: Front Public Health. 2023 Jun 15;11:1100335. doi: 10.3389/fpubh.2023.1100335 (PMC10309564; doi:10.3389/fpubh.2023.1100335)
Supplement: Supplementary file 1 [file Table_1.DOCX]

**Supplementary material**

**Supplementary Table 1.**Checklist for Reporting of Survey Studies (CROSS).

**Appendix 1.**The questionnaire with informed consent sent to the participants.

**Supplementary Table 1.**Checklist for Reporting of Survey Studies (CROSS).

| **Section/topic** | **Item** | **Description** | **Reported on page #** |
| --- | --- | --- | --- |
| **Title and abstract** | | |  |
| **Title and abstract** | 1a | State the word “survey” along with a commonly used term in title or abstract to introduce the study’s design. | 1 |
|  | 1b | Provide an informative summary in the abstract, covering background, objectives, methods, findings/results, interpretation/discussion, and conclusions. | 2,3 |
| **Introduction** | | |  |
| **Background** | 2 | Provide a background about the rationale of study, what has been previously done, and why this survey is needed. | 3,4 |
| **Purpose/aim** | 3 | Identify specific purposes, aims, goals, or objectives of the study. | 4 |
| **Methods** | | |  |
| **Study design** | 4 | Specify the study design in the methods section with a commonly used term (e.g., cross-sectional or longitudinal). | 4 |
|  | 5a | Describe the questionnaire (e.g., number of sections, number of questions, number and names of instruments used). | 4, 5 |
| **Data collection methods** | 5b | Describe all questionnaire instruments that were used in the survey to measure particular concepts. Report target population, reported validity and reliability information, scoring/classification procedure, and reference links (if any). | 4,5 |
|  | 5c | Provide information on pretesting of the questionnaire, if performed (in the article or in an online supplement). Report the method of pretesting, number of times questionnaire was pre-tested, number and demographics of participants used for pretesting, and the level of similarity of demographics between pre-testing participants and sample population. | 4,5 |
|  | 5d | Questionnaire, if possible, should be fully provided (in the article, or as appendices or as an online supplement). | Appendix 1 |
| **Sample characteristics** | 6a | Describe the study population (i.e., background, locations, eligibility criteria for participant inclusion in survey, exclusion criteria). | 4 |
|  | 6b | Describe the sampling techniques used (e.g., single stage or multistage sampling, simple random sampling, stratified sampling, cluster sampling, convenience sampling). Specify the locations of sample participants whenever clustered sampling was applied. | 4,5 |
|  | 6c | Provide information on sample size, along with details of sample size calculation. | 5 |
|  | 6d | Describe how representative the sample is of the study population (or target population if possible), particularly for population-based surveys. | 5 |
| **Survey**  **administration** | 7a | Provide information on modes of questionnaire administration, including the type and number of contacts, the location where the survey was conducted (e.g., outpatient room or by use of online tools, such as SurveyMonkey). | 4,5 |
|  | 7b | Provide information of survey’s time frame, such as periods of recruitment, exposure, and follow-up days. | 4,5 |
|  | 7c | Provide information on the entry process:  –>For non-web-based surveys, provide approaches to minimize human error in data entry.  –>For web-based surveys, provide approaches to prevent “multiple participation” of participants. | 4,5 |
| **Study preparation** | 8 | Describe any preparation process before conducting the survey (e.g., interviewers’ training process, advertising the survey). | 4 |
| **Ethical considerations** | 9a | Provide information on ethical approval for the survey if obtained, including informed consent, institutional review board [IRB] approval, Helsinki declaration, and good clinical practice [GCP] declaration (as appropriate). | 4, 12 |
|  | 9b | Provide information about survey anonymity and confidentiality and describe what mechanisms were used to protect unauthorized access. | 4, 12 |
| **Statistical analysis** | 10a | Describe statistical methods and analytical approach. Report the statistical software that was used for data analysis. | 5,6 |
|  | 10b | Report any modification of variables used in the analysis, along with reference (if available). | 5,6 |
|  | 10c | Report details about how missing data was handled. Include rate of missing items, missing data mechanism (i.e., missing completely at random [MCAR], missing at random [MAR] or missing not at random [MNAR]) and methods used to deal with missing data (e.g., multiple imputation). | NA |
|  | 10d | State how non-response error was addressed. | NA |
|  | 10e | For longitudinal surveys, state how loss to follow-up was addressed. | NA |
|  | 10f | Indicate whether any methods such as weighting of items or propensity scores have been used to adjust for non-representativeness of the sample. | NA |
|  | 10g | Describe any sensitivity analysis conducted. | NA |
| **Results** | | |  |
| **Respondent characteristics** | 11a | Report numbers of individuals at each stage of the study. Consider using a flow diagram, if possible. | 6,7 |
|  | 11b | Provide reasons for non-participation at each stage, if possible. | NA |
|  | 11c | Report response rate, present the definition of response rate or the formula used to calculate response rate. | 5 |
|  | 11d | Provide information to define how unique visitors are determined. Report number of unique visitors along with relevant proportions (e.g., view proportion, participation proportion, completion proportion). | 5,6,7 |
| **Descriptive**  **results** | 12 | Provide characteristics of study participants, as well as information on potential confounders and assessed outcomes. | 5,6,7 |
| **Main findings** | 13a | Give unadjusted estimates and, if applicable, confounder-adjusted estimates along with 95% confidence intervals and p-values. | NA |
|  | 13b | For multivariable analysis, provide information on the model building process, model fit statistics, and model assumptions (as appropriate). | 8 |
|  | 13c | Provide details about any sensitivity analysis performed. If there are considerable amount of missing data, report sensitivity analyses comparing the results of complete cases with that of the imputed dataset (if possible). | NA |
| **Discussion** | | |  |
| **Limitations** | 14 | Discuss the limitations of the study, considering sources of potential biases and imprecisions, such as non-representativeness of sample, study design, important uncontrolled confounders. | 11 |
| **Interpretations** | 15 | Give a cautious overall interpretation of results, based on potential biases and imprecisions and suggest areas for future research. | 8,9, 10 |
| **Generalizability** | 16 | Discuss the external validity of the results. | 8,9,10 |
| **Other sections** | | |  |
| **Role of funding source** | 17 | State whether any funding organization has had any roles in the survey’s design, implementation, and analysis. | 12 |
| **Conflict of interest** | 18 | Declare any potential conflict of interest. | 11 |
| **Acknowledgements** | 19 | Provide names of organizations/persons that are acknowledged along with their contribution to the research. | 12,13 |

**Appendix 1.** The questionnaire with informed consent sent to the participants.

**Knowledge, Attitude and Perception regarding COVID-19-Related Prevention Practice among Residents in Viet Nam: A cross-sectional Study.**

**Information Sheet and Informed Consent**

Dear Sir/Madam,

In the view of the number of COVID-19 infections spreading in Vietnam, especially the recent outbreak in Ho Chi Minh City, a research team from the Global COVID-19 Preventive Measures (<https://www.onlineresearchclub.org/covid-19-preventive-measures>) has been conducting a survey to assess the knowledge, attitudes, and practices towards to COVID-19 of residents in Ho Chi Minh City. This survey also evaluated the COVID effect on resident’s life. We believe that the information you provide could help improve the understanding of the risk of COVID-19 transmission and help prevent and control the pandemic.

In this survey, you will be asked to complete a questionnaire comprised of 21 questions, and it may take approximately 15-20 minutes of your time. Your participation in this study is voluntary. You are free to decline participation, leave blank any questions that you do not wish to answer, or withdraw from this study at any time. All of the responses in the survey will be recorded anonymously. The data collected will be kept confidential, and no one except the project team will access them. However, the results generated from the data will be disseminated through publication so that the scientific community, control programs and other concerned stakeholders can utilize them. We greatly appreciate your time and effort. For any further queries, you can contact our team by emailing the local researcher who invites you to participate and performs this survey.

Dr. Nguyen Tien Huy, Nagasaki University (Email: [tienhuy@nagasaki-u.ac.jp](mailto:tienhuy@nagasaki-u.ac.jp))

IMPORTANT NOTE: By completing and submitting this survey, you are indicating your consent to participate in the survey. There is no cost or reimbursement for participating in the study.

**Do you agree to participate in this research project?**

- **Yes**
- **No**

**Date: __ / __ /** 2021 **(**DD/MM/2021)

1. **DEMOGRAPHIC CHARACTERISTICS**
2. Age: …………..
3. Gender:

- Male
- Female
- Others, unspecified
- Others, please specify: ............................

1. Are you a medical staff?

- Yes
- No

1. Which resident locations are you living?

- Live in the center Ho Chi Minh city
- Live in suburban in Ho Chi Minh city (District 7, Nha Be, Can Gio, Binh Chanh, Binh Tan, District 12, Hoc Mon, Cu Chi)
- Thu Duc city (District 2, District 9, Thu Duc District)
- Others

1. What about your living accommodation?

- Private house
- Apartment
- Motel room
- Dormitory
- Others, please specify: ………….

1. What about your ethnicity?

- Kinh People
- Hoa People
- I don't want to answer
- Others

1. Do you have religious beliefs?

- Yes
- No

1. What about your current marital status?

- Single
- Married
- Divorce
- Separated
- Widowed
- Others

1. What about your education?

- Vocational training
- Elementary school
- Middle school
- High school
- University
- Postgraduation (Master Degree, PhD)
- Others

1. What about your current occupation?

- Full time
- Part time
- Unemployment
- Retirement
- Retirement
- Others, please specify: ........................

1. Which sources do you usually get information on COVID-19 pademic? *(multiple questions)*

- Television Channels (Yes/ No)
- Newspaper (Yes/ No)
- Local radio news (Yes/ No)
- Internet (Yes/ No)
- The Vietnamese Ministry of Health website (Yes/ No)
- Ho Chi Minh CDC website (Yes/ No)
- Others (Friends, Colleges, etc…)

**II. SURVEY ON COVID-19 KNOWLEDGE**

1. In your opinion, which is the COVID-19 transmission route? *(multiple answers)*

- The virus can be transmitted through small particles that produce as an infected person coughs, sneezes or talks.
- A person can get the virus when they touch a contaminated surface without to wash their hands, and then touch their eyes, nose, or mouth.
- Contact directly with the infected blood sample may be a route of transmission of the COVID-19 virus
- Others (please specify): ………………….

1. In your opinion, some symptoms of COVID-19 include:*( multiple answers)*

- Fever or chills (more common)
- Dry cough (more common)
- Fatigue (more common)
- Shortness of breath or difficulty breathing
- Sore throat
- Runny nose (Nasal discharge)
- Loss of smell (Anosmia)
- Loss of taste (Ageusia)
- Nausea, vomiting
- Headache
- Abdominal pain, diarrhea
- Others (please specify): ………………….

1. In your opinion, which of the following subjects is at high risk of severe pneumonia or death when infected with COVID-19? *(multiple answers)*

- Old age
- Children
- Underlying medical conditions(cardiovascular disease, chronic kidney disease, diabetes mellitus, ...)
- Obesity
- Underweight
- Unvaccinated against COVID-19 vaccine
- Newly vaccinated with 1 dose of COVID-19 vaccine
- Pregnant women
- I don’t know

1. In your opinion, are the asymptomatic individuals infected with COVID-19 at risk of spreading virus to people around them?

- Yes
- No
- I don’t know

**III. SURVEY ON COVID-19 ATTITUDES**

1. Please indicate your level of agreement with the following statements about some measures to prevent COVID-19 infection (From 9K message issued by the Vietnamese Ministry of Health):

|  | **Strongly disagree** | **Disagree** | **Neutral** | **Agree** | **Strongly agree** |
| --- | --- | --- | --- | --- | --- |
| 1. Keep your distance from others |  |  |  |  |  |
| 1. Always wear a facemask in public places, crowded areas, medical care or isolation areas |  |  |  |  |  |
| 1. Do not gather in large groups |  |  |  |  |  |
| 1. Always wash your hands with soap or hand sanitizer |  |  |  |  |  |
| 1. Clean daily the high-touch surfaces and objects (doorknobs, phones, tablets, tables, chairs, etc.) |  |  |  |  |  |
| 1. Keep the house clean, wash and keep the house with a good air flow |  |  |  |  |  |
| 1. Border Protection: Strengthen patrols, control entry into by land and sea road; strictly control, prevent and handle according to the law all illegally cross-border entry activities |  |  |  |  |  |
| 1. Isolation sites safely: Strengthen surveillance, compliance check for mandatory isolated processes, procedures and regulations in concentrated quarantine facilities. |  |  |  |  |  |
| 1. People were not allowed to leave their homes unless absolutely necessary; If they need to leave home, they must strictly follow the epidemic prevention and control policies. |  |  |  |  |  |
| 1. Do not public misinformation about COVID-19 caused confusion to the public's perception and impacts the effects of epidemic prevention and control from the Republican Party, the Government and the People. |  |  |  |  |  |

**IV. SURVEY ON COVID-19 PERCEPTIONS**

1. In your opinion, which of the following measures can help prevent the spread of COVID-19?

|  | **Yes** | **No** | **I don’t know** |
| --- | --- | --- | --- |
| 1. Avoid spending time in crowded areas |  |  |  |
| 1. Do not use public transportation |  |  |  |
| 1. Keep a distance of at least 2 meters from each other |  |  |  |
| 1. Use a private bathroom if it's possible |  |  |  |
| 1. Restricting contact with pets and other animals or always wash your hands thoroughly with soap and water before and after handling animals |  |  |  |
| 1. Wear a mask when there are people around |  |  |  |
| 1. Cover your nose and mouth when coughing or sneezing |  |  |  |
| 1. Avoid touching your face (eyes, nose, mouth) |  |  |  |
| 1. Always wash your hands |  |  |  |
| 1. Avoid sharing personal items with the others |  |  |  |
| 1. Clean daily the visibly dirty surfaces (such as phones, tablets, keyboards, remote controls, counters, countertops, bedside tables, doorknobs, bathroom fixtures, toilets). |  |  |  |

1. In your opinion, which of the following measures need to isolate at home for individuals who have been infected with COVID-19 (F0)? (*multiple answers*)

- Individual positively infected with COVID-19 should use a separate bedroom and bathroom, avoid close contact with all family members, and stay at least 2 meters or 6 feet (about 2 arm lengths) apart from others.
- If a patient has to share space, make sure the room has good air flow (open the windows, ...)
- Wear the facemask for both the sick and all family members.
- Just only wear a mask for the sick
- I don’t know

**V. SURVEY ON COVID-19 PRACTICE**

1. The following question examines what you have been doing in the past few days related to COVID-19 prevention. Please answer each issue in the question.

|  | **Yes** | **No** | **I don’t know** |
| --- | --- | --- | --- |
| 1. In recent days, have you been keeping your distance away from others? |  |  |  |
| 1. In recent days, do you often wear a face mask when going to public places, crowded areas, at medical facilities or isolation areas? |  |  |  |
| 1. In recent days, have you restricted going to crowded places? |  |  |  |
| 1. In recent days, have you limited communication with your neighbors? |  |  |  |
| 1. In recent days, do you often wash your hands with soap or hand sanitizer? |  |  |  |
| 1. In recent days, have you covered your nose and mouth when coughing or sneezing? |  |  |  |
| 1. In recent days, have you avoided touching your face (eyes, nose, and mouth)? |  |  |  |
| 1. In recent days, do you often clean the high-touch surfaces and objects (doorknobs, phones, tablets, tables, chairs…)? |  |  |  |
| 1. In recent days, do you often open the doors and windows, more frequently use the electric fans, and limit the use of air conditioners? |  |  |  |
| 1. In recent days, have you followed not to leave their homes unless absolutely necessary; If you need to leave home, you must strictly follow the epidemic prevention and control policies. |  |  |  |
| 1. Have you ever received two doses of the COVID-19 vaccine yet? |  |  |  |

1. In recent days, what difficulties do you have when applying isolated measures due to COVID-19 pandemic *(multiple answers)*

- Approach hardly to medical services (medical examination or follow-up visits)
- Income loss
- Job loss
- Not buying enough essential items (food, living items)
- Purchase hardly some necessary medicine
- Reduced income
- Reduced mental health
- Reduced physical health
- Raised in a family conflict
- Studying/working at home

1. In recent days, what difficulties do you have when applying the preventive measures of COVID-19 pandemic *(multiple answers)*

- Applied strategies for prevention of infections
- Changing habits
- Feeling unnecessary to apply infection prevention measures
- Get uncomfortable when taking infection prevention measures
- Shortage of personal protective equipment (facemask, hand sanitizer)
- Without any difficulty

THANK YOU FOR TAKING THIS SURVEY.
